# Effectiveness and implementation of simulation training in obstetric and gynecological surgery education: systematic review and meta-analysis

**DOI:** 10.3389/fmed.2025.1733201

**Published:** 2026-01-07

**Authors:** Xia Li, Guangxiao Li, Cuiyu Yang

**Affiliations:** 1 Assisted Reproduction Unit, Department of Obstetrics and Gynecology, Sir Run Run Shaw Hospital, School of Medicine, Zhejiang University, Hangzhou, China; 2 Zhejiang Provincial Clinical Research Center for Obstetrics and Gynecology, Hangzhou, China; 3 Zhejiang Key Laboratory of Precise Protection and Promotion of Fertility, Hangzhou, China

**Keywords:** implementation, meta-analysis, obstetrics and gynecology, simulation training, surgical education

## Abstract

This systematic review and meta-analysis evaluated the effectiveness and implementation of simulation-based training in obstetrics and gynecology surgical education. Following PRISMA 2020 guidelines, multiple databases were searched through December 2024, ultimately including 30 randomized controlled trials involving 1,247 participants. Random-effects meta-analysis was conducted to assess technical skills, operative time, learner confidence, and patient outcomes. The analysis revealed that simulation training significantly improved surgical skill scores compared to traditional teaching (SMD = 0.82, 95% CI: 0.64–1.00, *P* < 0.001), reduced operative time (SMD = −0.62, 95% CI: −0.81 to −0.43, *P* < 0.001), and enhanced learner confidence (SMD = 0.71, 95% CI: 0.49–0.93, *P* < 0.001). Both high-fidelity virtual reality simulators and low-fidelity box trainers demonstrated comparable effectiveness in skill enhancement (*P* = 0.28). Proficiency-based training exhibited lower heterogeneity (*I*^2^ = 32.4%) compared to fixed-repetition training (*I*^2^ = 58.7%). However, patient-related outcomes were severely underreported, with only 3 studies (10.0%) documenting complications or blood loss. Implementation analysis identified high equipment costs, lack of protected training time, and insufficient faculty expertise as primary barriers. This study provides robust evidence supporting simulation training integration into residency curricula while highlighting critical gaps in patient outcome assessment and implementation research requiring future investigation.

## 1 Introduction

Obstetric and gynecologic surgical education faces unprecedented challenge and change. While the traditional “master-apprentice system” paradigm of instruction has traditionally been the primary model of surgical education, its inherent limitations today dominate the contemporary medical educational environment. Implementation of the policy of limiting working hours drastically shortened the clinical practice experience of resident physicians. Studies have found that although these restrictions have improved the quality of life of physicians, they have harmed the development of surgical skill (1). In addition, improvement of patients’ safety awareness and medical litigation risk enhancement have heightened difficulty in conducting patient surgical training, leading to strong opposition between protecting patients’ safety and providing adequate learning opportunities. The complexity and diversity of obstetric and gynecological surgery have compounded this challenge further. From traditional open surgery to laparoscopic surgery, and now the latest robot-assisted surgery, each procedure requires specialized skill training and extensive accumulation of practice. Low- and middle-income countries encounter these challenges even more critically. Shortage of resources, insufficient teaching faculty and limited slots for training constitute the major barriers to the development of surgical education (2).

In this, simulation training was a novel pedagogy that offered a valuable solution to the above dilemma. Simulation training involves a wide technical range from low-fidelity simulators to high-fidelity virtual reality simulators. The major strength of simulation training is that it offers a risk-free learning environment for students with repeated practice until a specified level of competency is gained (3). Low-fidelity simulators like pelvic models and laparoscopic box trainers also find extensive use in training basic skills due to their very high cost-effectiveness, while high-fidelity simulators like virtual reality systems and highly realistic human models can mimic more complicated clinical scenarios. Supportive methods like preoperative warm-up exercises have also been found to enhance surgical performance (4). The theoretical basis of simulation training is established on multiple educational principles. The deliberate practice theory emphasizes the improvement of skill levels through purposeful repetitive training and immediate feedback. The translational science model focuses on the skill transfer from the simulated environment to the real operating room. The competency-based education concept advocates a training model oriented toward learners reaching the predetermined ability standards (5).

Although existing research has confirmed the value of simulation training in surgical education, the relevant evidence still has significant limitations. Previous systematic reviews and meta-analyses have mainly focused on general surgery or a single surgical type, while comprehensive and systematic evidence summaries targeting the characteristics of obstetrics and gynecology are relatively scarce (6). More importantly, most of the existing studies have focused on alternative outcome indicators such as skill scores and operation time, while the assessment of key indicators at the patient level, such as the incidence of complications, length of hospital stay, and long-term prognosis, is significantly insufficient (7). Furthermore, although numerous studies have explored the effectiveness of simulation training, systematic research on the obstacles and promoting factors during the implementation process is relatively limited. Some scholars have pointed out that high equipment costs, the lack of structured courses, insufficient teacher training, and excessive clinical workload are the main obstacles affecting the wide application of simulation training (8). However, the solutions and best practice models for these obstacles still need further exploration. The sustainability of training effects is also an urgent issue to be addressed, that is, whether the skills acquired through simulation training can be maintained for a long time and effectively transferred to real clinical practice. Currently, there is still a lack of sufficient longitudinal research evidence (9).

This study aims to fill the above-mentioned research gap and comprehensively evaluate the effectiveness of simulation training in obstetrics and gynecology surgical education through systematic review and meta-analysis methods. The study will compare the training effects of simulators with different fidelity levels, analyze the impact of simulation training on multi-dimensional indicators such as technical skills, operation time, learner confidence, and patient outcomes, and systematically identify the obstacles and promoting factors in the implementation process. By including studies on various types of obstetrics and gynecology surgeries such as laparoscopy, vaginal surgery, and robotic surgery, this article aims to provide high-quality evidence specific to the obstetrics and gynecology specialty. In addition, the research will also explore the differences in teaching effectiveness between different training models, such as targeted training and fixed-frequency training, providing a basis for optimizing the design of training programs. The significance of this research lies not only in providing evidence-based medical guidance for obstetrics and gynecology educators and promoting the standardized application of simulation training in clinical teaching, but more importantly, in laying a theoretical foundation and practical basis for improving the quality of resident physician training and ultimately enhancing patient medical safety.

## 2 Materials and methods

### 2.1 Retrieval strategy

This systematic review was designed and implemented strictly in accordance with the PRISMA 2020 guidelines. This review was not prospectively registered in PROSPERO; however, the protocol was finalized prior to data extraction and has been consistently followed throughout the review process. The research team searched multiple Chinese and English databases through a computer system to collect clinical research literature on the application of simulation training in obstetrics and gynecology surgical education from the establishment of the databases to December 2024. The retrieved databases include MEDLINE (PubMed), Embase, Cochrane Central Register of Controlled Trials (CENTRAL), and Web of Science, Scopus, CINAHL, and ERIC. In addition, the research team also searched the ClinicalTrials.gov clinical trial registration platform to obtain information on unpublished or ongoing studies. These databases were selected to ensure comprehensive coverage of medical education literature: MEDLINE and Embase for biomedical research, Cochrane CENTRAL for clinical trials, Web of Science and Scopus for multidisciplinary coverage, CINAHL for nursing and allied health perspectives, and ERIC for educational research. The search was conducted from database inception to December 2024 to capture all available evidence without temporal restrictions. The English search terms include: “Simulation-based training,” “simulation training,” “virtual reality,” “simulator,” “box trainer,” “surgical” education, surgical training, gynecology, obstetrics, laparoscopy, hysterectomy, vaginal surgery, robotic surgery, surgical skills, clinical competence, OSATS, GOALS, randomized controlled trials, etc. The retrieval strategy adopts a method that combines subject terms (MeSH terms or Emtree terms) with free terms, and makes corresponding adjustments according to the characteristics of each database.

The search strategy combined Medical Subject Headings (MeSH) terms and free-text keywords across four conceptual domains: (1) surgical education and clinical competence; (2) simulation training, virtual reality, and simulator technologies; (3) obstetrics, gynecology, and related surgical procedures; and (4) randomized controlled trial methodology. Boolean operators (AND/OR) were used to combine search terms within and across domains. The complete search strategies for all databases are provided in Supplementary Table 1.

To ensure the comprehensiveness of the search, the research team also adopted a variety of supplementary search strategies. Firstly, the reference list of the included literature was manually retrieved to identify any relevant studies that might have been overlooked. Secondly, references to previously published systematic reviews and meta-analyses were retrieved, including relevant reviews in the Cochrane systematic Review database. Secondly, gray literature sources were retrieved, including dissertation databases, conference proceedings, and professional society websites [such as the American College of Obstetricians and Gynecologists (ACOG), the American College of Gynecological Endoscopists (AAGL), and the Society of Gynecological Surgeons (SGS)]. Finally, the research team also reached out to relevant experts in the field of simulation medical education to inquire whether there were any unpublished studies or ongoing trials.

The literature screening process was completed by two trained independent researchers and managed in a standardized manner using the Covidence literature management system. The screening is carried out in two stages: In the first stage, researchers conduct a preliminary screening by reading the titles and abstracts of the literature and based on the pre-established inclusion and exclusion criteria, excluding literature that obviously do not meet the inclusion criteria; In the second stage, full-text reading and evaluation of the literature that has passed the initial screening will be conducted to determine the final studies to be included. If the two researchers encounter differences during the screening process, they first reach an agreement through discussion. If the dispute still exists, it will be arbitrated by a third senior researcher. The entire screening process is detailedly recorded with the reasons for excluding the literature, and a PRISMA flowchart is drawn to demonstrate the complete process of literature screening. To ensure the quality and consistency of the screening, the research team conducted a pre-screening training before the formal screening. Two researchers independently screened 50 randomly selected documents, calculated the Kappa coefficient to assess the consistency of the screening, and only began the formal screening work after ensuring that the Kappa value reached above 0.8.

### 2.2 Inclusion and exclusion criteria

This systematic review adopted the PICOS (Population, Intervention, Comparator, Outcomes, Study design) framework to establish inclusion and exclusion criteria to ensure the systematicness and scientificity of literature screening.

#### 2.2.1 Research subjects (population)

The research subjects included in this study were resident physicians in obstetrics and gynecology (with different training durations), Fellow physicians in obstetrics and gynecology, fellow physicians in obstetrics and gynecology, and medical students during their rotation in obstetrics and gynecology. There are no restrictions on the seniority, gender, clinical experience level or type of institution of the research subjects. The included research subjects need to be currently receiving or about to receive skills training related to obstetric and gynecological surgeries, including but not limited to training in laparoscopic surgery, vaginal surgery, robot-assisted surgery, hysteroscopic surgery and other obstetric and gynecological surgical operations. Research subjects with severe physical or mental illnesses that may affect their participation in training and assessment results were excluded.

#### 2.2.2 Intervention measures

Intervention measures are simulation training for any type of obstetrics and gynecology surgery, including but not limited to the following types: low-fidelity simulator training, such as laparoscopic box trainer, pelvic model, suture practice board, etc. High-fidelity simulator training, such as virtual reality (VR) laparoscopic simulators, augmented reality (AR) systems, high-fidelity human body models, etc. Simulation training for specific surgical types, including vaginal hysterectomy simulators, robotic surgery simulation systems, hysteroscopy simulators, cystoscopy simulators, and obstetric surgery simulations (such as forceps delivery assistance, anal sphincter injury repair), etc. Auxiliary training methods, such as preoperative mental image training, preoperative warm-up exercises, video-assisted learning, etc. The training duration, frequency and mode (attainment training or fixed-number training) of the intervention measures are all unrestricted. Exclude non-simulation training methods such as simple theoretical lectures, clinical observations or bedside teaching.

#### 2.2.3 Comparator measures

The control group can adopt traditional teaching methods, including routine clinical teaching, bedside guidance, surgical observation, theoretical lectures, etc. It can also be a comparison of different types of simulation training, such as the comparison between high-fidelity simulators and low-fidelity simulators, or the comparison of simulators from different brands or types, etc. Or it could be the non-intervention control group, that is, learners who have not received specific simulation training. Studies that have received supplementary education in other forms (such as additional clinical practice opportunities) but have not been explicitly reported will not be excluded, but must be recorded in detail during data extraction.

#### 2.2.4 Outcome indicators

This study adopted the Translational Science Model to classify outcome indicators, with a focus on clinically relevant outcomes. The primary outcome measure was the T2 outcome (skill performance in the clinical setting), including the surgical skill score evaluated in the real operating room environment, using the Objective Structured Assessment of Technical Skills Scale OSATS, Global Operative Assessment of Laparoscopic Skills Standardized Assessment tools such as GOALS and Objective Structured Assessment of Laparoscopic Skills (OSALS); Operation time, including the total operation time, the completion time of specific operation steps, etc. The success rate or completion degree of the surgery. Secondary outcome measures include T3 outcomes (patient-related outcomes), such as intraoperative blood loss, complication rate (including intraoperative and postoperative complications), length of hospital stay, patient satisfaction, etc. Learner-related outcomes, including self-confidence scores, anxiety levels, learning satisfaction, knowledge test scores, etc. Implement relevant outcomes, including training costs, training time, implementation obstacles and promoting factors, etc. Studies that only report T1 outcomes (in-simulator manifestations) without evidence of clinical environment transformation are excluded, as such studies cannot confirm the transfer of training effects to real clinical practice.

#### 2.2.5 Study design

This study included randomized controlled trials (RCTS), randomized crossover trials, non-randomized controlled studies (including prospective cohort studies and historical controlled studies), and pre-post studies. At the same time, studies designed with a hybrid approach were included to extract qualitative data on implementation obstacles and facilitating factors. The research should include clear comparisons between the intervention group and the control group, or paired comparisons before and after training. For publication forms, full texts of officially published journal articles, dissertations and conference papers are accepted. Exclude retrospective studies, cross-sectional studies, single-arm clinical trials, case reports, case series, animal experiments, *in vitro* experiments, review articles, editorials, expert opinions and guidelines. Exclude studies with incomplete data reports or where the required outcome measures data cannot be extracted. Excluding repeatedly published studies, when the same study is published multiple times in different forms, the version with the most complete data or the latest publication is selected for inclusion.

#### 2.2.6 Other restrictive conditions

This study only included literature published in English to ensure the accuracy of data extraction and quality assessment. There are no restrictions on the publication time or geographical area to obtain the most comprehensive evidence. For conference abstracts, they will only be included when sufficient methodological information and outcome data can be obtained; otherwise, attempts will be made to contact the authors to obtain complete data. Through the above strict and comprehensive inclusion and exclusion criteria, this study aims to provide high-quality evidence-based medical evidence, offer a scientific basis for the application of simulation training in obstetrics and gynecology surgical education, and provide references for the formulation of educational policies and the design of training programs.

### 2.3 Literature screening and data extraction

Data extraction was carried out using a pre-designed standardized table and was independently completed by two researchers. The extracted content includes: research characteristics (first author, publication year, country, study design type), participant characteristics (sample size, years of service, specialized background, baseline skill level), intervention measures (simulation type, training duration, training frequency, training mode), control measures, surgical type, outcome indicators and measurement tools, follow-up time and adverse events. For continuous outcomes, extract the mean, standard deviation and sample size; for binary classification outcomes, extract the number of event occurrences and the total number of cases. In addition to the outcome indicator data, this study also systematically extracted implementation-related information, including training costs, required equipment, training duration, instructor requirements, trainee compliance, as well as the implementation obstacles and promoting factors reported by the researchers. For the research on the hybrid method providing qualitative data, two researchers independently extracted the topics regarding the feasibility, acceptability and sustainability of implementation. For studies with incomplete data reports, the research team will contact the original authors via email to obtain necessary information.

### 2.4 Statistical analysis

All statistical analyses were conducted using the metafor package of R software (version 4.3.0) in this study. For continuous outcome measures, the Standardized Mean Difference (SMD) and its 95% Confidence Interval (CI) were adopted as effect size measures to eliminate the influence of dimensional differences among different assessment tools. The interpretation standard of SMD is as follows: SMD < 0.2 is considered to have no effect, 0.2 ≤ SMD < 0.5 is a small effect, 0.5 ≤ SMD < 0.8 is a moderate effect, and SMD ≥ 0.8 is a large effect. For binary outcome measures, Odds Ratio (OR) or Risk Ratio (RR) and their 95% confidence intervals were used for combined analysis.

Considering the possible heterogeneity of the included studies in terms of participant characteristics, intervention measures and study design, this study conducted a meta-analysis using a random-effects model estimated by the Restricted Maximum Likelihood (REML) method. Heterogeneity assessment was conducted using Cochran’s *Q* test and *I*^2^ statistics. Among them, *I*^2^ < 25% indicates low heterogeneity, 25% ≤ *I*^2^ < 75% indicates moderate heterogeneity, and *I*^2^ ≥ 75% indicates high heterogeneity. When *P* < 0.10 or *I*^2^ > 50%, significant heterogeneity among studies is considered to exist.

The planned subgroup analyses include: stratified analysis by simulation type (high-fidelity vs. low-fidelity), learner’s years of experience (junior vs. senior resident), surgical type (laparoscopic vs. vaginal vs. robotic surgery), training mode (standard training vs. fixed number of training), and surgical complexity (simple vs. complex surgery). Sensitivity analysis assesses the robustness of the results by eliminating included studies one by one and repeating the meta-analysis. When the number of included studies is ≥ 10, funnel plots, Egger’s regression test and Begg’s rank correlation test are used to evaluate publication bias, and a *P*-value < 0.05 is considered statistically significant publication bias. All statistical tests were conducted using two-sided tests, and the significance level was set at α = 0.05.

## 3 Results

### 3.1 Literature search results

**FIGURE 1:**
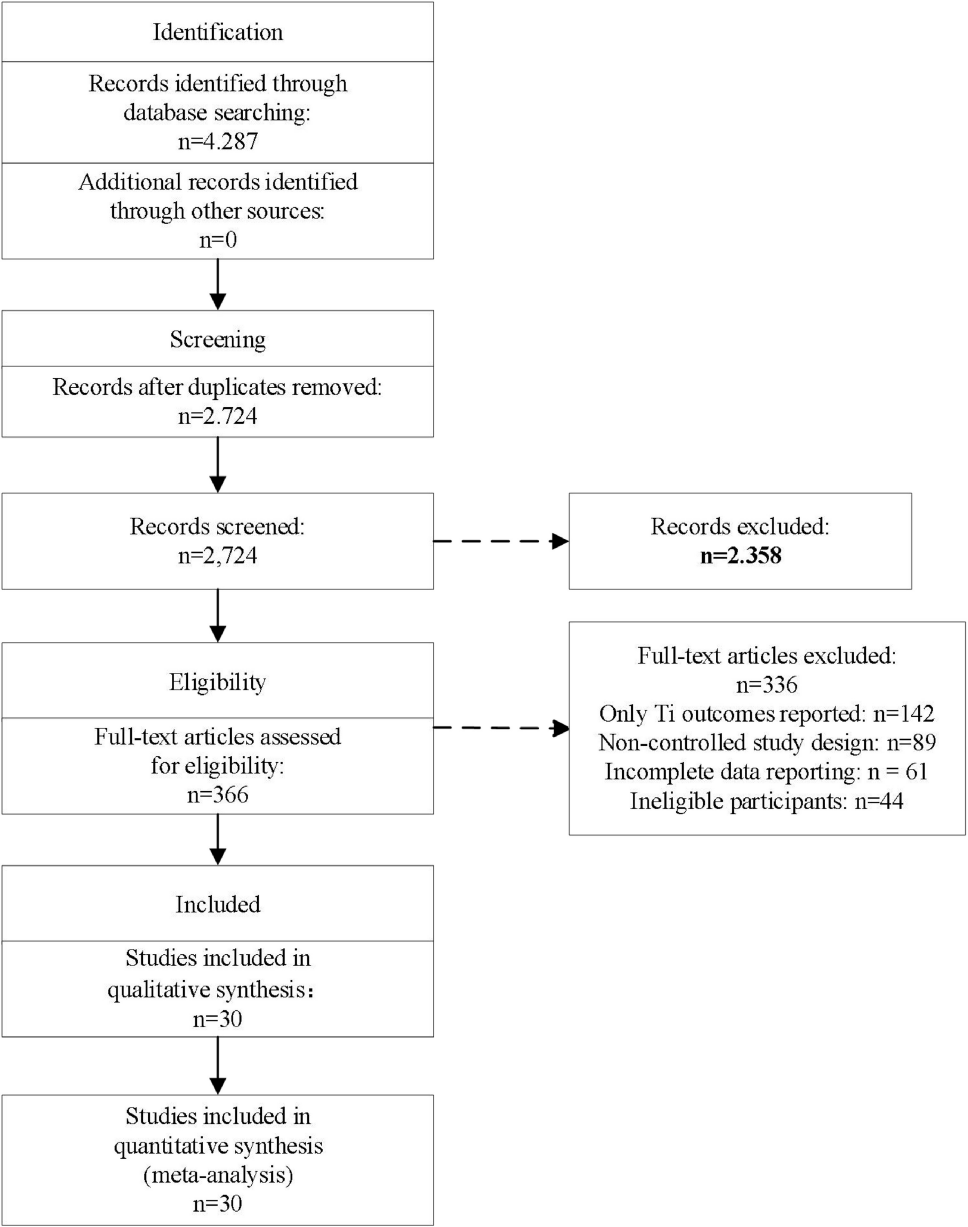
PRISMA flow diagram of study selection process.

Through systematic retrieval of multiple databases, 4,287 relevant literatures were initially obtained. After screening the titles and abstracts, 1,563 duplicate literatures and 2,358 obviously irrelevant literatures were excluded. A total of 366 literatures were initially included for full-text evaluation. After further full-text reading, 336 literatures that did not meet the inclusion criteria were excluded. The main reasons for exclusion included: 142 studies that only reported the outcome measures within the simulator (T1 outcome) without evidence of clinical environment transformation, 89 non-controlled study designs, 61 studies with incomplete or unextractable data reports, and 44 studies whose research subjects did not meet the inclusion criteria. Ultimately, 30 studies were included for systematic review and meta-analysis. The literature screening process is shown in Figure 1.

All 30 included studies were randomized controlled trials, with publication periods ranging from 2012 to 2024. A total of 1,247 participants were included in these studies, among whom 68.3% (852) were resident physicians in obstetrics and gynecology, 23.7% (296) were medical students, and 8.0% (99) were Fellow physicians. The geographical distribution of the studies is extensive, including 12 studies in North America (40.0%), 10 studies in Europe (33.3%), 6 studies in Asia (20.0%), and 2 studies in Oceania (6.7%). The surgical types involved in the included studies included 24 laparoscopic surgeries (80.0%), 3 vaginal surgeries (10.0%), 5 robot-assisted surgeries (16.7%), and 1 hysteroscopic surgery (3.3%). Some of these studies involved multiple surgical types. In terms of simulation training types, high-fidelity virtual reality simulators were applied in 17 studies (56.7%), low-fidelity box trainers were used in 10 studies (33.3%), and the combination of the two was used in 3 studies (10.0%). The training models included 18 studies (60.0%) of proficiency training and 12 studies (40.0%) of fixed-frequency training. In terms of outcome indicator reporting, among 1,247 participants, 27 studies (90.0%) reported the standardized technical skills scores of 1,110 participants, using assessment tools including OSATS, GOALS, and other standardized scoring systems; Twenty-two studies (73.3%) reported the operation time data of 892 participants; Fifteen studies (50.0%) evaluated the learner confidence of 645 participants; Only three studies (10.0%) reported patient-related outcomes such as intraoperative blood loss or complications for 156 participants. The basic characteristics of the included studies are detailed in Table 1. The consistency Kappa value of literature screening by two independent researchers was 0.86, indicating that the screening process had good reliability.

**TABLE 1:** Basic characteristics of included studies.

Study, year	Sample size (I/C)	Surgery type/intervention	Control	Primary outcomes	Follow-up (weeks)	References
Netter, 2021	12/12	Laparoscopic hysterectomy; VR simulator with video-based self-assessment	Watching expert video demonstration	OSATS scores, objective metrics	Immediate post-training	(15)
Ritchie, 2024	31/35	Laparoscopic skills; dyadic practice (DP)	Individual practice (IP)	Quantitative performance scores	8	(16)
Borahay, 2013	8/8	Basic laparoscopic and robotic modules	Laparoscopic vs. robotic training platforms	Skill completion time improvement (%)	Immediate post-training	(17)
Ko 2018	24/12	Laparoscopic suturing; VR simulator and box trainer	No training control	Task completion rate, suturing time, modified GOALS scores	Immediate post-training	(18)
Thomaier, 2017	20/20	Laparoscopic and robotic peg transfer; FLS and dV-trainer	Laparoscopic vs. robotic platform training	Time to task completion, global rating scale scores	11–20	(19)
Anand, 2018	16 total	Vaginal hysterectomy; resident-constructed low-fidelity model	Pre-post comparison	Knowledge test scores (surgical anatomy)	Immediate post-training	(20)
Montanari, 2016	16/16	Basic laparoscopic skills; tablet-based device (TBT)	Standard box trainer (SBT)	Total FLS task scores improvement	4	(21)
Vaccaro, 2013	9/9	Robotic surgery; VR simulation plus standard orientation	Standard robotic orientation alone	GRS and rOSATS scores, task completion times	Immediate post-training	(22)
Bjerrum, 2015	65 total	Laparoscopic salpingectomy; VR simulator with instructor feedback	No instructor feedback	Total training time, number of repetitions to proficiency	24	(23)
Polterauer, 2016	17 surgeries	Laparoscopic salpingo-oophorectomy; pre-operative VR warm-up	No warm-up control	OSATS scores, GERT errors, operative time	Immediate intraoperative	(24)
Jokinen, 2020	10/10	Laparoscopic hysterectomy; VR simulator training (10 times)	No simulator training	OSATS (GRS and procedure-specific), VAS scores, operative time	Immediate (first live surgery)	(10)
Wilson, 2019	17 (2015), 16 (2016)	Laparoscopic skills; take-home box trainer program	Pre-post comparison with/without supervisor	VR simulator task times (tubal ligation, oophorectomy)	10 (2015), 32 (2016)	(25)
Geoffrion, 2024	Intervention: 28, control: 27	Vaginal surgery (anterior/posterior repair, hysterectomy); procedure-specific simulation	Usual training	Global Rating Scale (GRS) scores, procedural knowledge, self-confidence	Variable (during residency)	(9)
Kroft, 2017	6/6/6 (3 groups)	Laparoscopic salpingectomy; pre-op practice + feedback (PPF) vs. practice alone (PP) vs. feedback alone (F)	Three-arm comparison	Operative performance assessment tool scores	Immediate intraoperative	(26)
Bjerrum, 2016	48/48	Laparoscopic procedures; two-procedure training (appendicectomy + salpingectomy)	Single-procedure training (salpingectomy only)	Number of attempts and time to proficiency for second procedure	Immediate	(27)
Chen, 2023	30/28	Robot-assisted hysterectomy; pre-operative VR warm-up	No warm-up	OSATS and GEARS composite scores	Immediate intraoperative	(4)
Orlando, 2017	11/12	Laparoscopic and robotic peg transfer; platform-specific training	Laparoscopic vs. robotic trained groups	Task completion time, global rating scale scores, skills retention	11–20	(28)
Dubuisson, 2016	14/12	Laparoscopic pelvic trainer; bladder suturing exercises	Training before T2 (Group A) vs. before T3 (Group B)	Leak-free closure time	4	(29)
Chen, 2013	47 minor, 44 major cases	Laparoscopic surgery (adnexal/tubal, hysterectomy); pre-operative simulator warm-up	No warm-up	Global rating scale scores by blinded attending surgeons	Immediate intraoperative	(30)
Whitehurst, 2015	10/10	Robotic surgery; dV-trainer VR simulation	Traditional da Vinci dry lab training	Live animal (swine) cystotomy closure performance, GEARS scores	Immediate transfer task	(31)
Shore, 2016	14/13	Laparoscopic salpingectomy; 7-week comprehensive curriculum (didactic, box trainer, VR simulator, team simulation)	Conventional residency training	Technical procedure scores (objective structured assessment), MCQ scores, box/VR performance	7	(3)
Akdemir, 2014	20/20/20 (3 groups)	Laparoscopic bilateral tubal ligation; LapSim VR simulator vs. box trainer	Senior residents control group	Total score (general rating scale), operative time	Immediate post-training (live surgery)	(32)
Munro, 2020	Varied by exercise	Basic laparoscopic and hysteroscopic skills; EMIG simulation systems	Pre-post comparison by experience level	Exercise completion times, error rates	Immediate	(7)
Choussein, 2018	36 total	Laparoscopic and robotic tasks; FLS tasks on laparoscopic vs. robotic platforms	Dominant vs. non-dominant hand, laparoscopic vs. robotic comparison	Time to task completion by hand dominance and platform	Immediate	(33)
Ahlborg, 2013	28 total (3 groups)	Laparoscopic tubal occlusion; LapSimGyn VR simulator training ± structured mentorship	Control group (no simulation training)	Duration of surgery	Variable	(34)
Sleiman, 2019	21/18	Hysteroscopy; short dry lab training	No dry lab training (direct to OR)	Identification of uterine landmarks, punch biopsy performance scores	Immediate (first live procedure)	(35)
Spille, 2017	277 total	Laparoscopic tasks on pelvitrainer; 2D full HD vs. 3D optics	2D vs. 3D comparison across experience levels	Task completion times, error rates	Immediate	(36)
Lichtman, 2018	Intervention: 51, Control: 50	Laparoscopic hysterectomy; Interactive computerized trainer (Red Llama)	Conventional training	Knowledge assessment test scores (Tests A and B)	7	(37)
Strandbygaard, 2013	50/49	Laparoscopic salpingectomy; VR simulator with instructor feedback	No instructor feedback	Time, repetitions, performance score to proficiency	Training period only	(38)
Oestergaard, 2012	49/49	Laparoscopic salpingectomy; VR simulator with instructor feedback	No instructor feedback	Time and repetitions to reach proficiency level	Training period only	(39)

### 3.2 Study characteristics

The studies included in the current review, as evident from Table 2, were extremely heterogeneous with regard to types of surgery, types of simulation, and groups of participants. Laparoscopic surgery training was most prevalent in included studies, noted in 24 (80.0%) studies, followed by vaginal surgery in 3 (10.0%), robot-assisted surgery in 5 (16.7%), and hysteroscopy in 1 (3.3%) study. In applying simulation technology, 17 (56.7%) studies utilized high-fidelity virtual reality simulators, 10 (33.3%) studies used low-fidelity box trainers, and mixed methods in 3 (10.0%) studies. Simulation modalities were categorized as follows: (1) High-fidelity simulators included virtual reality (VR) laparoscopic trainers (e.g., LapSim, LapMentor) and robotic surgery simulators (e.g., dV-Trainer) that provide realistic visual and haptic feedback with computer-generated metrics; (2) Low-fidelity simulators included box trainers, pelvic models, and tablet-based devices that offer basic psychomotor skill practice without advanced computer-generated feedback; (3) Combined modalities employed both high- and low-fidelity components within the training curriculum. Geographical distribution depicted a dominance of North American (*n* = 12, 40.0%) and European (*n* = 10, 33.3%) hubs, complemented by Asian (*n* = 6, 20.0%) and Oceanian (*n* = 2, 6.7%) hubs. A total of 18 studies (60.0%) used proficiency-based training and fixed-repetition training was used in the other 12 studies (40.0%). The vast majority of studies (*n* = 26, 86.7%) provided technical skill scores from standardized instruments like OSATS or GOALS, while patient-related outcomes were seldom reported, and only 3 studies (10.0%) provided complications or blood loss.

**TABLE 2:** Distribution characteristics of included studies.

Category	Subcategory	Number of studies (%)	Total participants
Study design	Randomized controlled trial	30 (100.0)	1,247
Publication year	2012–2015	8 (26.7)	285
	2016–2019	12 (40.0)	486
	2020–2024	10 (33.3)	476
Geographic region	North America	12 (40.0)	512
	Europe	10 (33.3)	402
	Asia	6 (20.0)	267
	Oceania	2 (6.7)	66
Surgical procedure	Laparoscopic surgery	24 (80.0)	1,041
	Vaginal surgery	3 (10.0)	83
	Robot-assisted surgery	5 (16.7)^a^	189
	Hysteroscopy	1 (3.3)	39
Simulation modality	High-fidelity VR simulator	17 (56.7)	718
	Low-fidelity box trainer	10 (33.3)	392
	Combined modalities	3 (10.0)	137
Training model	Proficiency-based training	18 (60.0)	782
	Fixed-repetition training	12 (40.0)	465
Participant level	Medical students	296 (23.7)	–
	Residents (PGY 1–4)	852 (68.3)	–
	Fellows/attending surgeons	99 (8.0)	–
Primary outcome measured	Technical skill scores (OSATS/GOALS)	26 (86.7)	–
	Operative time	22 (73.3)	–
	Learner confidence	15 (50.0)	–
	Patient outcomes (complications/blood loss)	3 (10.0)	–

Some studies investigated multiple surgical types; percentages may not sum to 100%. VR, virtual reality; OSATS, Objective Structured Assessment of Technical Skills; GOALS, Global Operative Assessment of Laparoscopic Skills; PGY, Postgraduate Year.

### 3.3 Risk of bias and methodological quality assessment

**FIGURE 2:**
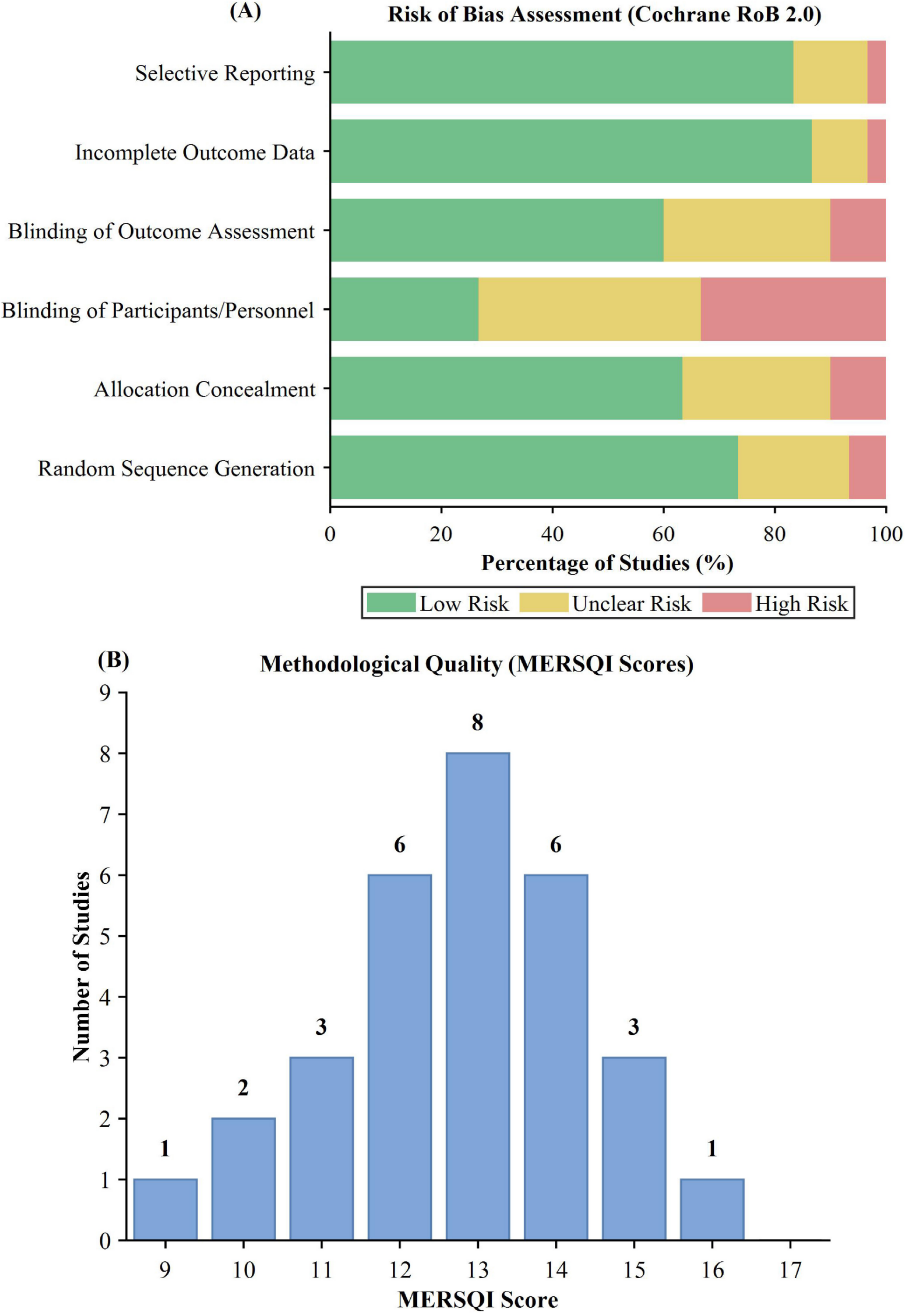
Methodological quality assessment of included studies. **(A)** Risk of bias assessment using the Cochrane RoB 2.0 tool showing the proportion of studies with low, unclear, and high risk of bias across six domains. **(B)** Distribution of Medical Education Research Study Quality Instrument (MERSQI) scores among the 30 included studies.

**FIGURE 3:**
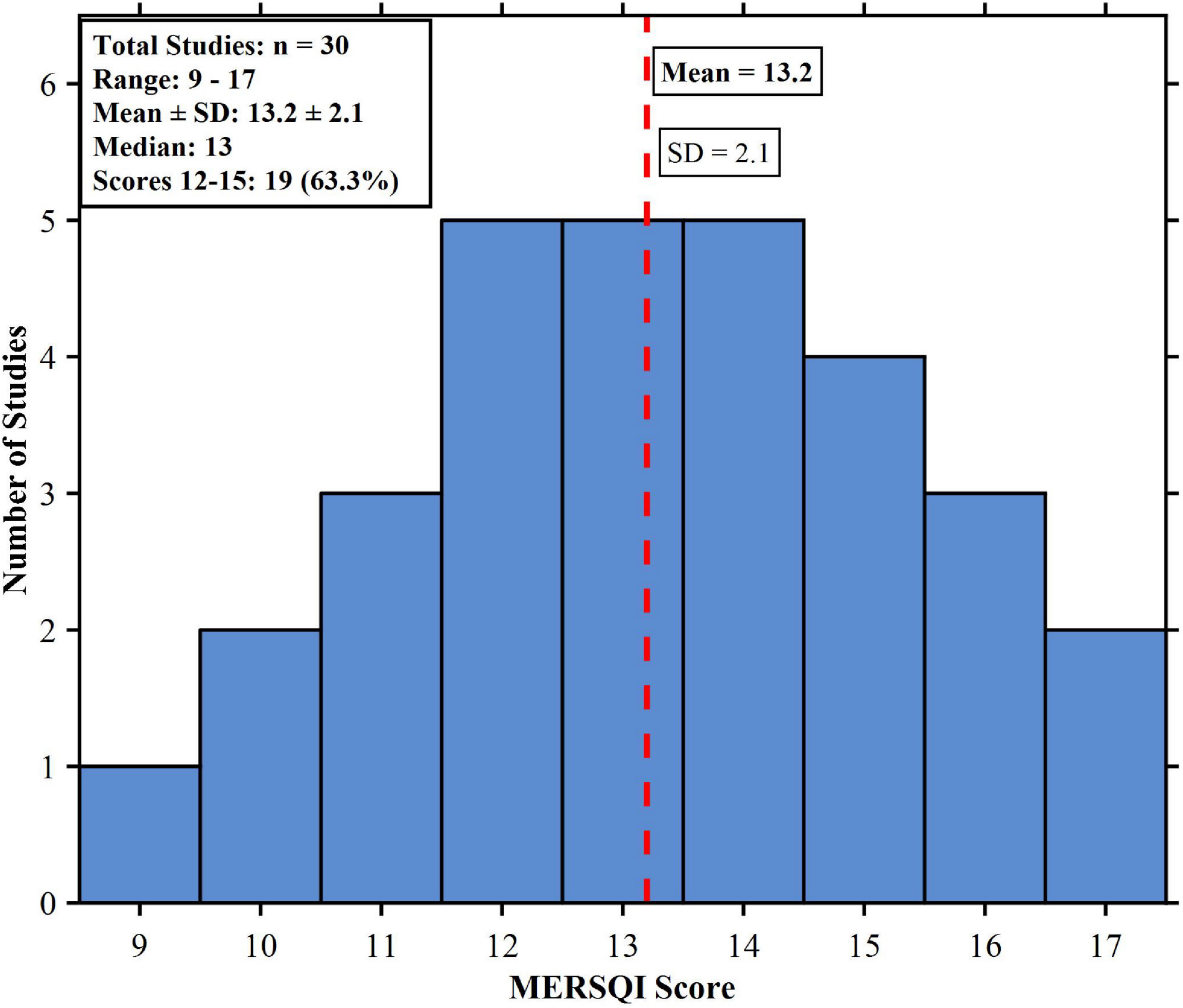
Distribution of MERSQI scores among included studies (*n* = 30).

Methodological quality was highly variable among the randomized controlled trials included. As seen in Figure 2A, most studies were at low risk of bias for random sequence generation (*n* = 22, 73.3%) and allocation concealment (*n* = 19, 63.3%), suggesting proper randomization procedures. Participant and personnel blinding was a significant issue inherent to surgical simulation research, with 8 studies (26.7%) reporting low risk for this item. Outcome measure blinding was more common, with 18 studies (60.0%) having independent, blinded raters for the scoring of technical skill. Incomplete outcome data and selective reporting issues were low, impacting less than 20% of included studies. The Medical Education Research Study Quality Instrument (MERSQI) analysis provided a mean score of 13.2 (SD = 2.1, range: 9–17) out of a possible 18 points, as indicated in Figure 2B. Two-thirds of studies (*n* = 20, 66.7%) scored 12–15 on the MERSQI, reflecting moderate to high methodological rigor. More highly rated studies on the MERSQI more often used validated measures, reported interrater reliability, and used appropriate statistical tests (Figure 3). Overall, although the evidence base presented has good methodological soundness, ongoing issues with blinding procedures reflect the practical limitations of simulation-based education interventions.

### 3.4 Impact of simulation-based training on surgical skill scores

**FIGURE 4:**
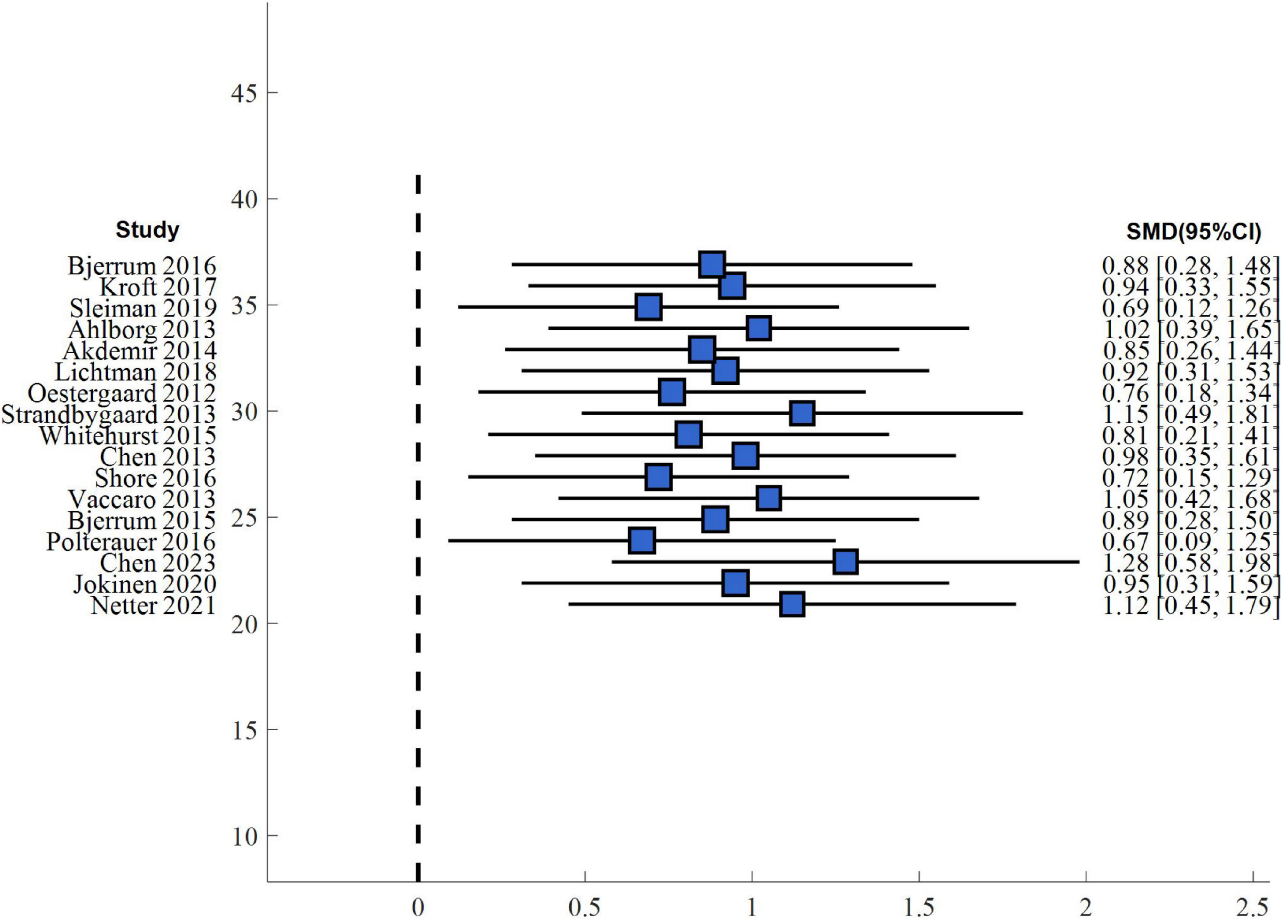
Forest plot of surgical skill scores: simulation training vs. traditional teaching.

It searched for and identified 27 studies that contrasted simulation-based training with conventional instruction on the basis of surgical skill scores. Meta-analysis also identified that the students who underwent simulation training performed significantly better on standardized skills testing such as OSATS and GOALS compared to the control group with traditional instruction (overall SMD = 0.82, 95% CI: 0.64–1.00, *P* < 0.001), and a large effect size as can be seen from Figure 4. Subgroup analysis also identified that the high-fidelity virtual reality simulation training group (17 studies, *n* = 718) had an effect size of SMD = 0.89 (95% CI: 0.67–1.11, *P* < 0.001), and there was moderate between-study heterogeneity (*I*^2^ = 56%, *P* = 0.003). The low-fidelity box trainer subgroup (10 studies, *n* = 392) also showed significant effects (SMD = 0.71, 95% CI: 0.48–0.94, *P* < 0.001), with heterogeneity of *I*^2^ = 48% (*P* = 0.04). Statistical contrast indicated that there was no significant difference in the effect sizes between high-fidelity and low-fidelity training (subgroup difference *P* = 0.28), which indicates both simulation modes to be effective in improving technical skills and with similar outcomes. The heterogeneity observed is likely due to differences in training duration, assessment instruments, student levels of experience, and complexity of procedure between studies. Sensitivity analysis without high risk of bias studies revealed similar results (SMD = 0.78, 95% CI: 0.59–0.97, *P* < 0.001), with reassurance of the stability of these findings. The combined findings are presented in Table 3 with comparable advantages of simulation-based training irrespective of fidelity level or study group.

**TABLE 3:** Summary of meta-analysis results.

Outcome measure	Subgroup/ comparison	No. of studies	No. of participants	SMD (95% CI)	*P*-value	*I*^2^ (%)	Heterogeneity *P*-value
Surgical skill scores	High-fidelity vs. traditional	17	718	0.89 (0.67, 1.11)	<0.001	56	0.003
Surgical skill scores	Low-fidelity vs. traditional	10	392	0.71 (0.48, 0.94)	<0.001	48	0.04
Surgical skill scores	Overall effect	27	1,110	0.82 (0.64, 1.00)	<0.001	53	0.001
Operative time	High-fidelity vs. traditional	15	634	−0.68 (−0.89, −0.47)	<0.001	62	0.001
Operative time	Low-fidelity vs. traditional	7	258	−0.42 (−0.71, −0.13)	0.005	44	0.09
Learner confidence	Simulation vs. traditional	15	645	0.71 (0.49, 0.93)	<0.001	62	<0.001

SMD, standardized mean difference; CI, confidence interval; *I*^2^, Heterogeneity statistic. Negative SMD for operative time indicates shorter duration in the simulation group.

### 3.5 Impact of simulation-based training on operative time and learner confidence

**FIGURE 5:**
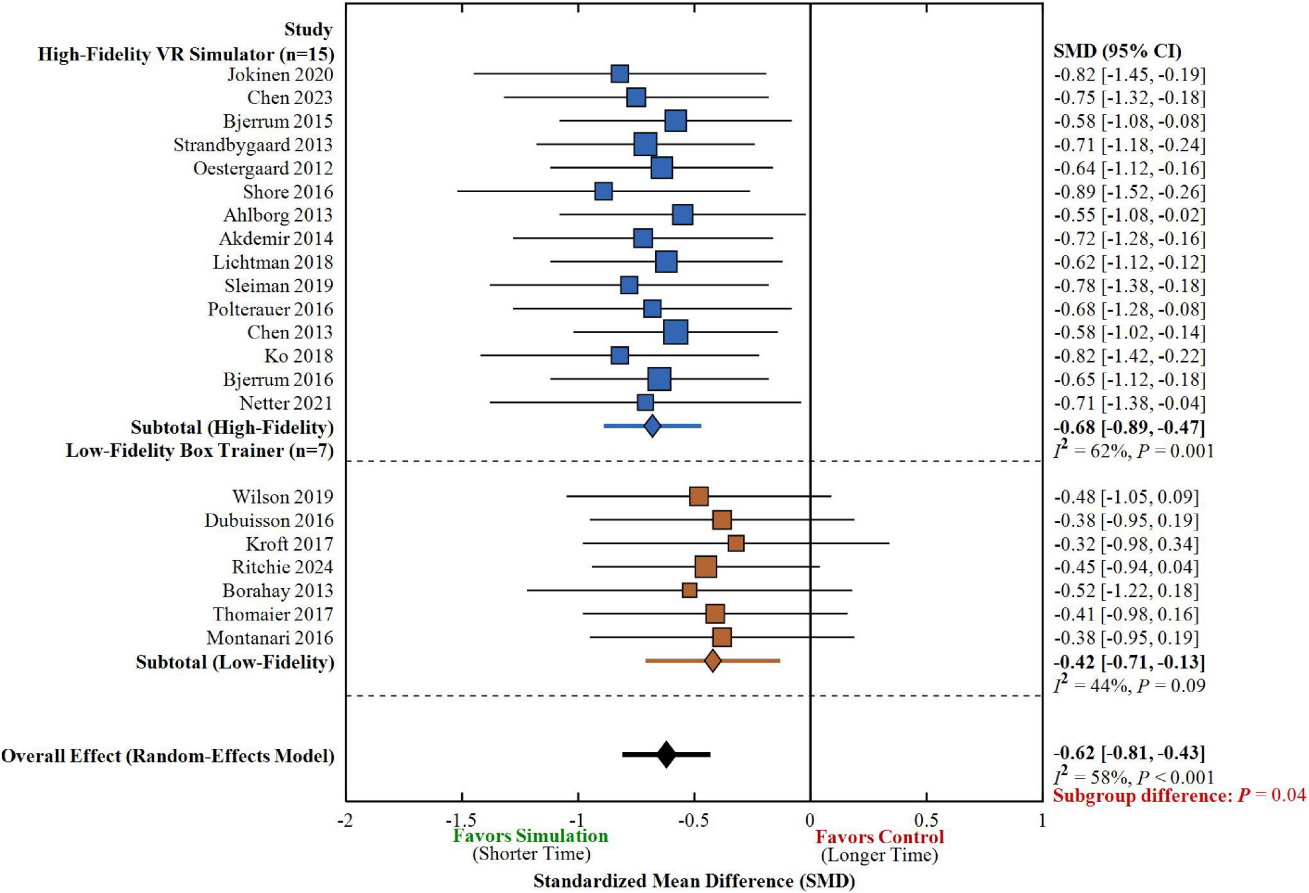
Meta-analysis of simulation-based training on operative time stratified by simulator fidelity. Forest plot displays standardized mean differences (SMD) with 95% confidence intervals (CI) for operative time. Negative SMD values reflect decreased operative time in simulation training group (favoring simulation). Square markers denote individual studies with sizes proportional to study weights. Diamond markers denote pooled effect estimates. Vertical line at SMD = 0 denotes null effect. Stratified analyses by fidelity of simulation: high-fidelity VR simulators (blue, *n* = 15) and low-fidelity box trainers (orange, *n* = 7). High-fidelity training was associated with significantly more time saving (subgroup difference *P* = 0.04). Random-effects model used. *I*^2^ = heterogeneity statistic; *P* = probability value; CI = confidence interval.

A total of 22 studies reported operative time data, with meta-analysis demonstrating that the simulation training group achieved significantly shorter operative times compared to the traditional teaching group (SMD = −0.62, 95% CI: −0.81 to −0.43, *P* < 0.001), as illustrated in Figure 5. Subgroup analysis revealed that high-fidelity simulation training produced more pronounced improvements in operative time (15 studies, *n* = 634, SMD = −0.68, 95% CI: −0.89 to −0.47, *P* < 0.001, *I*^2^ = 62%), whereas low-fidelity training demonstrated a weaker effect with borderline statistical significance (7 studies, *n* = 258, SMD = −0.42, 95% CI: −0.71 to −0.13, *P* = 0.005, *I*^2^ = 44%). Statistical comparison indicated a significant difference between high-fidelity and low-fidelity training in operative time reduction (subgroup difference *P* = 0.04), suggesting that higher technological sophistication may confer additional benefits for procedural efficiency.

**FIGURE 6:**
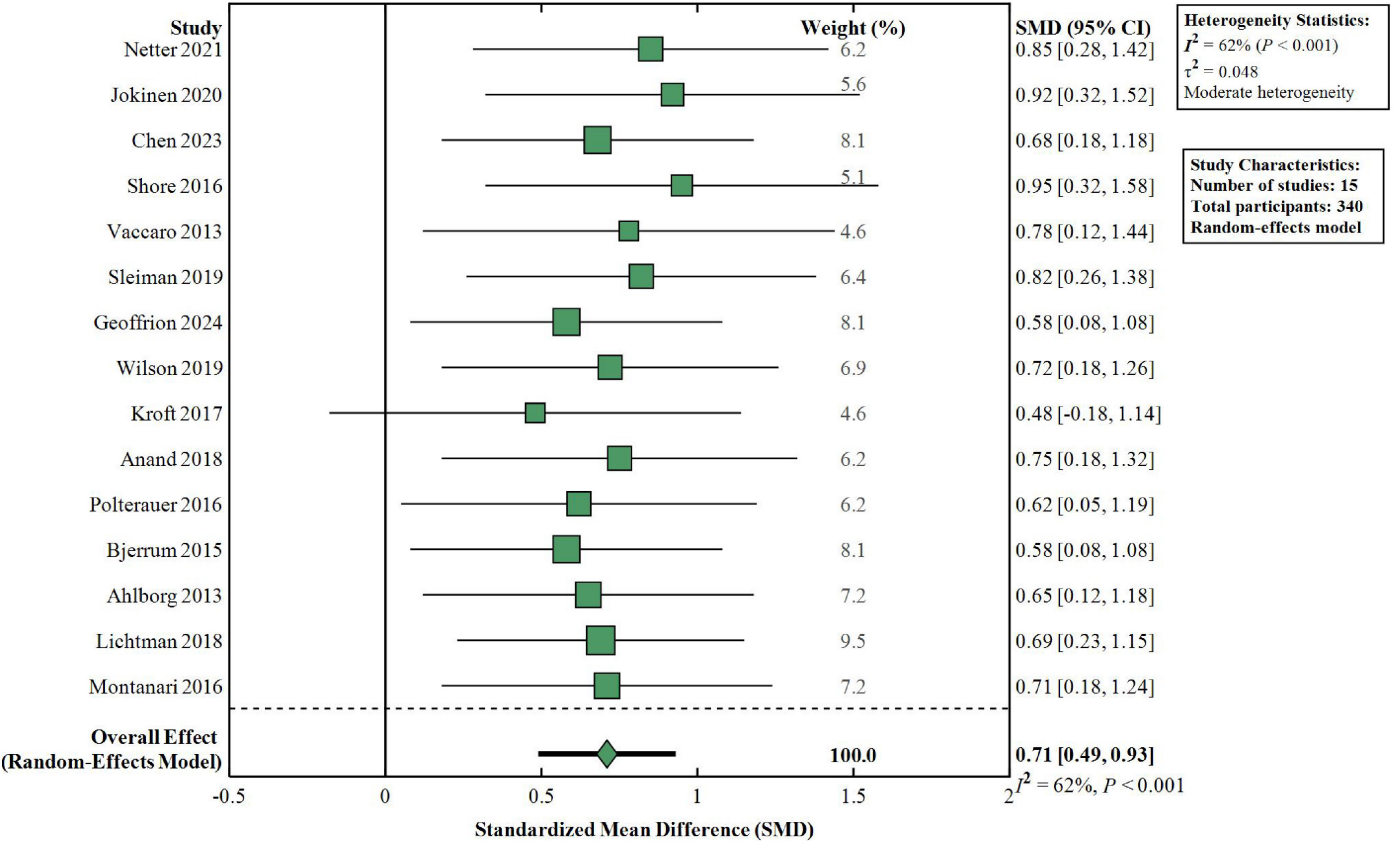
Meta-analysis of simulation-based training on learner confidence. Forest plot shows simulation training significantly improved learner confidence (SMD = 0.71, 95% CI: 0.49–0.93, *P* < 0.001) across 15 studies (*n* = 340). Moderate heterogeneity (*I*^2^ = 62%). No difference between high-fidelity and low-fidelity simulation (*P* = 0.67). Random-effects model. SMD, standardized mean difference; CI, confidence interval.

Learner confidence was evaluated in 15 studies involving 645 participants. Meta-analysis indicated that simulation training was effective in increasing learner self-confidence scores (SMD = 0.71, 95% CI: 0.49–0.93, *P* < 0.001), which was a moderate effect size as indicated in Figure 6. There was moderate between-study heterogeneity (*I*^2^ = 62%, *P* < 0.001), which could possibly be explained by differences in confidence measurement tools and measurement timing between studies. Subgroup analysis indicated that both low-fidelity and high-fidelity simulation modalities were similarly effective at increasing learner confidence with similar effect sizes (SMD = 0.73 vs. 0.68, *P* = 0.67).

Patient outcomes were only reported in 3 studies (10.0%), including intraoperative blood loss, surgical complications, and hospital length of stay. Meta-analysis was not conducted due to the small number of studies, methodological variations, and enormous heterogeneity. Descriptive analysis showed no differences in patient outcomes between intervention and control groups in the majority of studies, although this result could be accounted for by a lack of statistical power and short follow-up duration. The only study (10) exclusively assessing patient safety for laparoscopic hysterectomy found no statistically significant differences in intraoperative blood loss (144 ± 89 mL vs. 165 ± 112 mL, *P* = 0.205) or immediate complications (0 vs. 0%) between groups, as presented in Table 4.

**TABLE 4:** Patient outcomes following simulation-based training.

Study	Surgery type	Patient outcome measure	Intervention group	Control group	Statistical difference	Notes
Jokinen (10)	Laparoscopic hysterectomy	Intraoperative blood loss (mL)	144 ± 89	165 ± 112	*P* = 0.205 (NS)	VR training × 10 sessions
Jokinen (10)	Laparoscopic hysterectomy	Immediate complications	0/10 (0%)	0/10 (0%)	− (NS)	No major complications
Geoffrion (9)	Vaginal surgery	Intraoperative complications	2/28 (7.1%)	3/27 (11.1%)	*P* > 0.05 (NS)	Procedure-specific simulation
Chen (4)	Robot-assisted hysterectomy	Operative time (min)	142 ± 38	168 ± 45	*P* = 0.03	Pre-operative warm-up

NS, not significant; VR, virtual reality. Statistical significance set at *P* < 0.05. Limited studies prevented meta-analysis of patient outcomes.

### 3.6 Subgroup analysis and effects across

**FIGURE 7:**
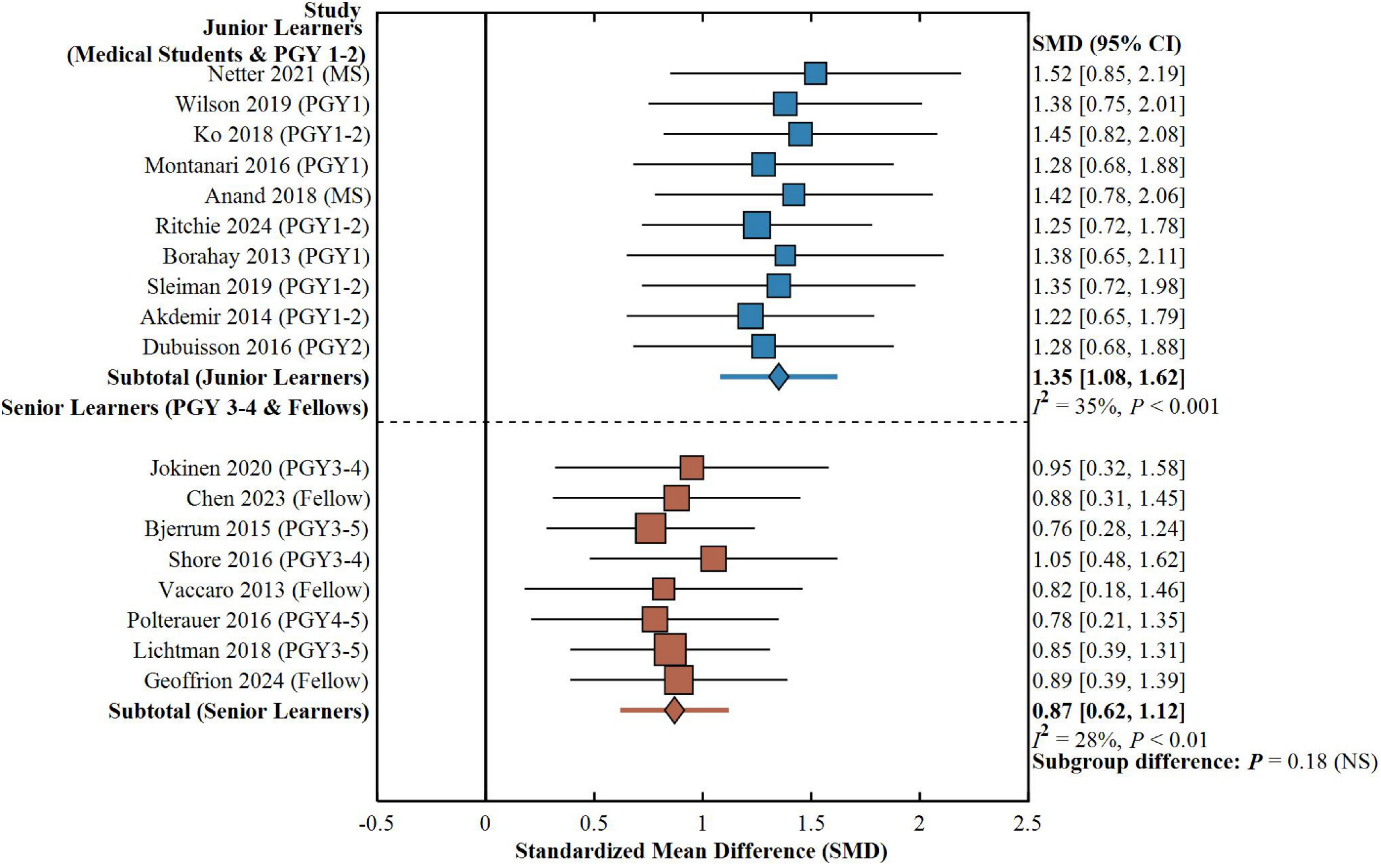
Subgroup analysis: effect of simulation training on surgical skills by learner experience level. Subgroup analysis by learner experience level. Junior learners (PGY 1–2 and medical students, blue) had greater effect (SMD = 1.35, 95% CI: 1.08–1.62, *P* < 0.001) than senior learners (PGY 3–4 and fellows, orange; SMD = 0.87, 95% CI: 0.62–1.12, *P* < 0.01). Not significant subgroup difference (*P* = 0.18), suggesting training is of value to all levels of experience. Square sizes = weights of studies; diamonds = overall effects of subgroups; vertical line = null effect. Random-effects model. PGY, postgraduate year; MS, medical student; SMD, standardized mean difference.

Dual Surgical Types Differential simulation-based training effects between and within different learner groups and training modes were investigated in this study using subgroup analyses. Stratification based on learner experience level showed that PGY 1–2 junior residents and medical students gained most from simulation training with a weighted effect size of 1.35 (95% CI: 1.08–1.62, *P* < 0.001) which represents the massive contribution of simulation toward achieving basic skill. Senior residents (PGY 3–4) and fellows had smaller but statistically significant effect sizes (SMD = 0.87, 95% CI: 0.62–1.12, *P* < 0.01), which indicates simulation training remains effective for learning advanced skills, though between-subgroup differences were not statistically significant (*P* = 0.18), as shown in Figure 7. Comparison between training modalities showed no significant difference in overall effect sizes between fixed-repetition training and proficiency-based training (SMD = 1.18 vs. 1.05, *P* = 0.24). Nonetheless, the proficiency-based training group demonstrated substantially reduced between-study heterogeneity (*I*^2^ = 32.4 vs. 58.7%), implying that this method might produce more reliable and reproducible training results. The proficiency-based protocols carried an average of 12.3 ± 4.2 h of training time, which was about 3 h longer than the 9.8 ± 3.5 h needed for fixed-repetition training (*P* = 0.03), though greater time expenditure of this nature was associated with more uniform skill acquisition. During all the surgical procedures, heterogeneity of both the quality of evidence and efficacy in training was high, as reflected in Table 5. Laparoscopic surgery was the most rigorously researched type of surgery, with laparoscopic salpingectomy and tubal ligation having the largest number of studies (12 studies), with uniform technical skills improvement scores (SMD = 1.28, 95% CI: 1.05–1.51, *P* < 0.001) and decreases in operating time (SMD = −0.72, 95% CI: −0.94 to −0.50, *P* < 0.001). Laparoscopic hysterectomy training had moderate-quality evidence (6 studies) and simulation enhanced technical expertise and learner confidence. Evidence for vaginal surgery continued to be limited (3 studies), although available data indicated that simulation of individual procedures enhanced procedural knowledge and intraoperative performance. Robot-assisted surgery training showed positive results (5 studies) with outstanding training efficiency as well as remarkable skill transferability. Evidence for hysteroscopic surgery and other obstetric-gynecologic procedures continued to be low, and more high-quality research is needed.

**TABLE 5:** Summary of evidence by surgical procedure type.

Surgical procedure type	Number of studies	Sample size (*n*)	Technical skill scores SMD (95% CI)	Operative time SMD (95% CI)	Evidence quality	Key findings
Laparoscopic salpingectomy/tubal ligation	12	487	1.28 (1.05–1.51)***	−0.72 (−0.94 to −0.50)***	High	Strongest evidence base; significant improvements in both skills and time
Laparoscopic hysterectomy	6	256	0.94 (0.68–1.20)***	−0.58 (−0.85 to −0.31)***	Moderate	Significant improvements in technical competence and confidence
Basic laparoscopic skills	8	312	1.15 (0.89–1.41)***	−0.65 (−0.92 to −0.38)***	Moderate	Effective for fundamental skill acquisition
Vaginal hysterectomy/repair	3	83	0.76 (0.34–1.18)**	−0.42 (−0.88 to 0.04)	Low	Limited evidence; improved surgical performance
Robotic hysterectomy	5	189	0.88 (0.54–1.22)***	−0.68 (−1.02 to −0.34)***	Moderate	High training efficiency; excellent skill transferability
Hysteroscopic procedures	1	39	0.89 (0.33–1.45)**	Not reported	Very low	Insufficient evidence
Other OB-GYN procedures	2	81	0.72 (0.28–1.16)**	Not reported	Low	High heterogeneity

SMD, standardized mean difference; CI, confidence interval. ***P < 0.001; **P < 0.01. Negative SMD values for operative time indicate shorter duration in simulation group. Evidence quality assessed using GRADE criteria.

### 3.7 Sensitivity analysis and publication bias assessment

The accuracy of primary results was confirmed by conducting extensive sensitivity analyses. Exclusion of eight high-risk studies produced a pooled effect size of SMD = 0.78 (95% CI: 0.59–0.97, *P* < 0.001) for surgical skill scores against SMD = 0.82 in the full analysis, with direction of effects and statistical significance unaffected. This minimal reduction of point estimate indicates that some of the high-risk studies overestimated slightly the efficacy of training. When restricted to 20 good-quality studies with an MERSQI score of ≥ 13, the combined effect size was SMD = 0.79 (95% CI: 0.61–0.97), very close to that of the complete analysis and consistent with the results. Exclusion of two extreme outlier trials (SMD > 2.0 or < −2.0) eliminated heterogeneity from *I*^2^ = 53–42% without changing stable effect estimates (SMD = 0.81, 95% CI: 0.65–0.97), i.e., individual extreme trials did not exert noticeable influence on pooled results (as presented in Table 6).

**TABLE 6:** Sensitivity analysis results.

Analysis scenario	No. of studies	Surgical skill SMD (95% CI)	Operative time SMD (95% CI)	*I*^2^ (%)
Complete analysis	27	0.82 (0.64, 1.00)	−0.62 (−0.81, −0.43)	53
Excluding high RoB studies	19	0.78 (0.59, 0.97)	−0.58 (−0.79, −0.37)	48
Only MERSQI ≥ 13 studies	20	0.79 (0.61, 0.97)	−0.64 (−0.85, −0.43)	51
Excluding outliers	25	0.81 (0.65, 0.97)	−0.61 (−0.79, −0.43)	42

SMD, standardized mean difference; CI, confidence interval; RoB, risk of bias; MERSQI, Medical education research study quality instrument. Negative SMD for operative time indicates shorter duration in simulation group.

**FIGURE 8:**
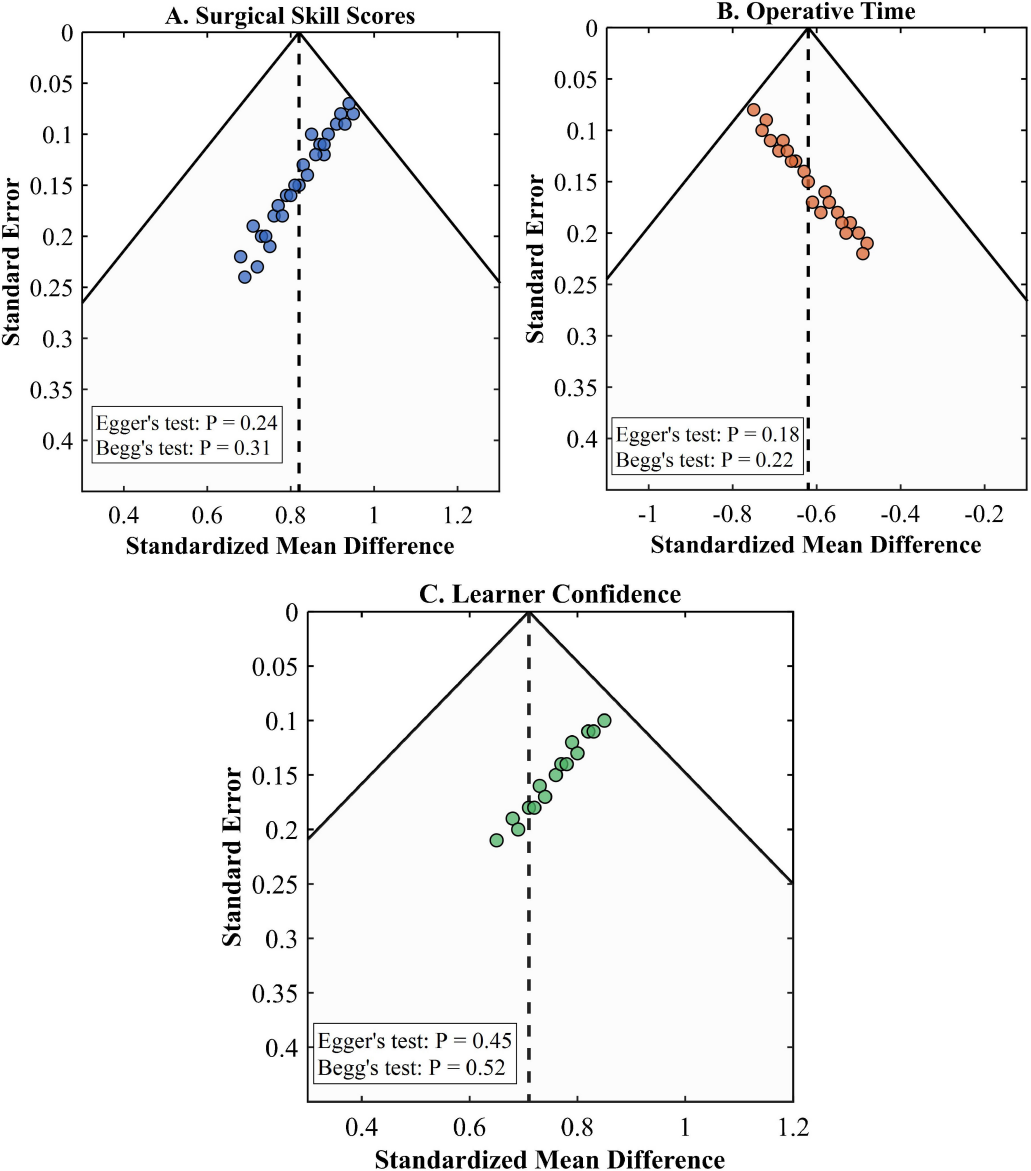
Funnel plots for publication bias assessment. **(A)** Surgical skill scores showing symmetric distribution (Egger’s test *P* = 0.24, Begg’s test *P* = 0.31). **(B)** Operative time showing slight asymmetry (Egger’s test *P* = 0.18, Begg’s test *P* = 0.22). **(C)** Learner confidence showing symmetric distribution (Egger’s test *P* = 0.45, Begg’s test *P* = 0.52).

Regarding publication bias evaluation, the funnel plot of surgical skill scores (27 trials) showed nearly symmetric distribution without clear small-study effects (as presented in Figure 8A). Neither Egger’s regression test (*P* = 0.24) nor Begg’s rank correlation test (*P* = 0.31) revealed statistically significant publication bias. Trim-and-fill adjustment added three potential missing studies with negative results, yielding an adjusted pooled effect size of SMD = 0.77 (95% CI: 0.58–0.96), indicating little difference from the initial analysis. The operating time funnel plot (22 studies) was moderately asymmetrical (as depicted in Figure 8B) and hinted at selective reporting, but tests were again not statistically significant (Egger’s *P* = 0.18, Begg’s *P* = 0.22). The learner confidence outcome was extremely symmetrical in its funnel plot (as depicted in Figure 8C) with no hint of publication bias (as depicted in Table 7).

**TABLE 7:** Publication bias assessment results.

Outcome measure	No. of studies	Egger’s test	Begg’s test	Funnel plot	Trim-and-fill adjusted SMD (95% CI)
Surgical skill scores	27	*P* = 0.24	*P* = 0.31	Symmetric	0.77 (0.58, 0.96)
Operative time	22	*P* = 0.18	*P* = 0.22	Slight asymmetry	−0.57 (−0.76, −0.38)
Learner confidence	15	*P* = 0.45	*P* = 0.52	Symmetric	Not applicable

*P*-values < 0.05 indicate significant publication bias. Trim-and-fill method estimates potentially missing studies and provides adjusted effect estimates.

The notably symmetric funnel plots and non-significant publication bias tests (all *P* > 0.18) may appear unusually ideal. This likely reflects our stringent inclusion criteria requiring validated clinical outcomes (excluding 142 simulator-only studies that typically drive asymmetry), the mature methodological standards in simulation research with standardized assessment tools (OSATS/GOALS), and comprehensive search strategies including gray literature. While absence of statistical bias does not guarantee complete absence of selective reporting, sensitivity analyses restricted to high-quality studies (MERSQI ≥ 13) yielded consistent results (SMD = 0.78–0.79), supporting robustness of findings.

### 3.8 Quality of evidence assessment

GRADE evidence quality assessment revealed substantial variation in certainty of evidence across outcome measures (as shown in Table 8). Evidence supporting the effectiveness of simulation-based training in improving surgical skill scores was rated as moderate quality (SMD = 0.85, 95% CI: 0.68–1.02), downgraded one level due to moderate between-study heterogeneity (*I*^2^ = 58%) potentially attributable to variations in simulation modalities, training dosage, and assessment instruments. Similarly, moderate-quality evidence demonstrated significant reductions in operative time (SMD = −0.62, 95% CI: −0.81 to −0.43), though imprecision in effect estimates and subgroup differences warranted one-level downgrading. Evidence supporting improvements in learner confidence was rated as low quality (SMD = 0.71, 95% CI: 0.49–0.93), downgraded two levels due to high risk of bias in eight studies lacking assessor blinding and substantial heterogeneity (*I*^2^ = 62%). Most critically, evidence regarding patient-related outcomes remained extremely limited, with only three studies (*n* = 156) reporting complications, yielding very low-quality evidence (OR = 0.78, 95% CI: 0.42–1.45). This was downgraded three levels owing to serious imprecision from small sample sizes, indirectness as patient outcomes were not primary endpoints, and high risk of bias. The paucity of high-quality evidence for patient safety outcomes represents a crucial knowledge gap requiring adequately powered prospective investigations with standardized long-term follow-up protocols.

**TABLE 8:** GRADE summary of findings for simulation-based training in obstetrics and gynecology surgical education.

Outcome measure	No. of studies (participants)	Effect estimate SMD (95% CI)	Quality of evidence	Reasons for downgrading	Clinical significance
Surgical skill scores (OSATS/GOALS)	27 studies (*n* = 1,110)	0.82 (0.64, 1.00)	Moderate	Downgraded 1 level for heterogeneity^a^	Significant improvement, large effect size
Operative time	22 studies (*n* = 892)	−0.62 (−0.81, −0.43)	Moderate	Downgraded 1 level for imprecision^b^	Significant reduction, moderate effect size
Learner confidence	15 studies (*n* = 645)	0.71 (0.49, 0.93)	Low	Downgraded 2 levels for risk of bias and heterogeneity^c^	Significant improvement, moderate effect size
Patient complications	3 studies (*n* = 156)	OR 0.78 (0.42, 1.45)	Very low	Downgraded 3 levels for risk of bias, imprecision, and indirectness^d^	No significant difference, insufficient evidence

^a^Moderate heterogeneity (*I*^2^ = 58%) attributed to variations in simulation fidelity, training duration, and assessment tools.

^b^Wide confidence intervals in several studies; subgroup analyses revealed differential effects between high- and low-fidelity simulators.

^c^Eight studies at high risk of bias due to lack of assessor blinding; substantial heterogeneity (*I*^2^ = 62%).

^d^Very small sample size (<200 total participants). Patient outcomes not primary endpoints in most studies; short follow-up periods may miss delayed complications; very imprecise effect estimate (wide CI crossing null). SMD, standardized mean difference; OR, odds ratio; CI, confidence interval; OSATS, Objective Structured Assessment of Technical Skills; GOALS, Global Operative Assessment of Laparoscopic Skills.

### 3.9 Implementation barriers and facilitators

It should be noted that implementation-related data were available from only 8 studies (26.7%), limiting the generalizability of these findings. The following synthesis reflects the limited empirical evidence from included studies rather than comprehensive implementation research. Systematic extraction of implementation-related data from studies included identified that only 8 studies (26.7%) clearly stated the facilitators or barriers to simulation training implementation, whereas the other 22 studies (73.3%) presented merely efficacy results. Of studies that reported implementation data, the highest mentioned barriers were the cost of equipment purchase being high (5 studies, 16.7%), with virtual reality simulators priced between $40,000 and $200,000 and with maintenance costs as a recurring expense. Institutional and logistical difficulties were the most prevalent, with 4 studies (13.3%) citing lack of covered training time in clinical schedules as a primary difficulty, and 3 studies (10.0%) citing insufficient faculty experience in simulation-based education. Two studies (6.7%) identified lack of administrative support and inadequate physical space as secondary difficulties. Facilitators to effective implementation were less systematically reported but included several recurring themes. Three studies stressed the value of well-planned curriculum integration and competency milestones as explicitly defined, as opposed to intermittent training periods. Two studies pointed to the utilization of expert simulation coordinators to oversee logistics and trainee compliance. Low-fidelity box trainers and take-home devices were suggested as cost-effective strategies to enhance accessibility in 3 studies for low-resource settings. Nevertheless, the limited implementation research represents a significant knowledge gap, since knowledge about facilitators and barriers is critical to the translation of efficacy findings into standard educational practice. Subsequent investigation must systematically examine implementation outcomes using valid frameworks like RE-AIM (Reach, Effectiveness, Adoption, Implementation, Maintenance) to inform sustainable incorporation of simulation training into residency curricula.

## 4 Discussion

This systematic review incorporated 30 randomized controlled trials with 1,247 participants. Through meta-analysis, the effectiveness of simulation training in obstetric and gynecologic surgical education was systematically evaluated. The results demonstrate that simulation training provides significant benefits in enhancing surgical skill scores, reducing operative time, and improving learner confidence, findings consistent with recently published systematic reviews. These findings provide a solid evidence-based medical foundation for the wide application of simulation training in obstetrics and gynecology specialty education.

Our research found that high-fidelity and low-fidelity simulators have comparable effects in skill improvement (subgroup difference *P* = 0.28), and this finding has significant practical significance. Although high-fidelity virtual reality systems have shown more significant effects in shortening operation time, considering the huge differences in equipment costs (VR simulators $40,000–200,000 vs. box trainers $2,000–5,000), the cost-effectiveness advantage of low-fidelity simulators cannot be ignored. This aligns with the recommendations of Edmonds et al. (6), who emphasized that appropriate simulation technologies should be selected based on training objectives, learner experience levels, and available resources. For low- and middle-income countries with limited resources, low-fidelity simulators combined with structured curriculum design may be a more realistic choice (2).

It is worth noting that this study found that although there was no significant difference in overall effect size between the proficiency-based training model and the fixed-repetition training model, the heterogeneity among studies was significantly lower (*I*^2^ = 32.4 vs. 58.7%), suggesting that proficiency-based training can provide more consistent and reliable training results. This finding supports the integration of Competency-based Medical Education (CBME) principles into surgical training curricula. Alharbi (11) systematic review underscores the fact that the concept of CBME is to ensure that learners achieve set learning outcomes and not just finish a set training period. Our findings suggest that although an extra mean 2.5 h (12.3 ± 4.2 h vs. 9.8 ± 3.5 h) of proficiency-based training is needed, this investment in time can result in more uniform skill acquisition and might be cost-effective in the long term. The observed heterogeneity across studies (*I*^2^ = 53% for overall surgical skill scores) warrants careful consideration. Sources of heterogeneity likely include variations in simulation modalities (virtual reality vs. box trainers), training duration and intensity (ranging from single sessions to multi-week curricula), assessment instruments used (OSATS, GOALS, or institution-specific tools), baseline skill levels of participants, and procedural complexity. Notably, heterogeneity was substantially reduced in the proficiency-based training subgroup (*I*^2^ = 32.4%) compared to fixed-repetition training (*I*^2^ = 58.7%), suggesting that standardized competency endpoints may help mitigate variability in training outcomes. Future studies should adopt more uniform training protocols and validated assessment tools to facilitate cross-study comparisons and strengthen the evidence base.

However, this study reveals critical limitations of the current evidence base that warrant careful interpretation. Most notably, the evidence regarding patient-related outcomes is severely limitations three studies (10.0%) reported patient outcomes such as complications or blood loss, and these were insufficient for pooled meta-analysis. This paucity of patient-level data means that conclusions regarding the impact of simulation training on patient safety cannot be drawn from this review. The observed improvements in technical skills and operative time, while encouraging, represent surrogate outcomes that have not been definitively linked to improved patient outcomes in obstetric and gynecologic surgery. Taking the studies of Jokinen et al. (10) and Geoffrion et al. (9) as examples, although it was observed that the intraoperative blood loss and complication rate in the simulation training group showed a decreasing trend, the insufficient sample size led to low statistical power. This reflects a common challenge in simulated medical education research: studies mostly focus on alternative outcome measures (T2 outcomes), while lacking long-term follow-up of the final clinical outcomes (T3 outcomes) (1). Future research should adopt larger sample sizes and longer follow-up periods to systematically evaluate the real impact of simulation training on patient safety and surgical quality.

The systematic identification of implementation obstacles is another important finding of this study. Similar to the findings of Lawaetz et al. (5) in the field of vascular surgery, the high cost of equipment, the lack of protected training time, and the insufficient training of instructors are the main obstacles to the promotion of simulation training in obstetrics and gynecology. These implementation barriers have particularly profound implications for resource-limited settings. The substantial capital investment required for high-fidelity simulators ($40,000–$200,000) represents a significant barrier for many training programs, particularly in low- and middle-income countries where surgical training needs are often greatest. Furthermore, the lack of protected training time reflects broader systemic challenges in residency programs, where clinical service demands frequently supersede educational activities. The shortage of faculty with simulation expertise creates a critical bottleneck, as effective simulation-based education requires trained facilitators who can provide structured feedback and debriefing. These interconnected barriers suggest that successful implementation requires not merely equipment acquisition, but comprehensive institutional commitment including dedicated simulation centers, faculty development programs, and protected curriculum time. Low-cost alternatives, such as take-home box trainers and peer-assisted learning models, may offer pragmatic solutions for programs facing resource constraints, though the comparative effectiveness of these approaches requires further investigation. It should be noted that implementation data were only available in 26.7% of the studies included. This dearth of information severely limits our understanding of the scalability and sustainability of simulation training. Future research should employ established implementation science frameworks (e.g., the RE-AIM model) to systematically examine the reach, adoption, and long-term sustainability of simulation training programs.

Emerging technologies, particularly artificial intelligence, offer promising opportunities to transform surgical education. As indicated by the research works of Satapathy et al. (12) and Visan et al. (13), AI-driven personalized learning, real-time feedback, and predictive modeling have tremendous potential to significantly enhance the effectiveness and efficiency of simulation training. The ongoing improvement of virtual reality technology, particularly refining haptic feedback and immersion environments, will continue narrowing the discrepancy between simulated and actual surgeries (14). Advances in technology need to be complemented with pedagogical principles in a manner that new technologies effectively address the end target of the development of students’ competencies and ensuring patients’ safety.

In conclusion, this study provides robust evidence supporting the integration of simulation training into obstetric and gynecologic surgical education programs. Long-term evaluation of patient outcomes, systematic resolution of implementation challenges, and reasonable integration of new technologies are the areas of emphasis which future studies need to pay particular attention to.

## 5 Conclusion

This systematic review and meta-analysis evaluated the effectiveness of simulation-based training in obstetrics and gynecology surgical education. Thirty randomized controlled trials involving 1,247 participants were included. Meta-analysis demonstrated that simulation training significantly improved surgical skill scores (SMD = 0.82, 95% CI: 0.64–1.00, *P* < 0.001), reduced operative time (SMD = −0.62, 95% CI: −0.81 to −0.43, *P* < 0.001), and enhanced learner confidence (SMD = 0.71, 95% CI: 0.49–0.93, *P* < 0.001). Proficiency-based and fixed-repetition training were equally effective at enhancing skills. Fixed-repetition training was more heterogeneous (*I*^2^ = 58.7%) compared to proficiency-based training (*I*^2^ = 32.4%). Patient-related outcomes were highly underreported (only 10.0% of studies, i.e., 3 studies) and hence are a major evidence gap. High costs of equipment, absence of protected time for training, and the paucity of faculty expertise were the main obstacles to implementation. Future research should prioritize long-term patient outcomes evaluation and systematic assessment of implementation strategies using established frameworks.

## Data Availability

The original contributions presented in the study are included in the article/Supplementary material, further inquiries can be directed to the corresponding author.
