# Supplementary material for: Effectiveness and implementation of simulation training in obstetric and gynecological surgery education: systematic review and meta-analysis
Source: Front Med (Lausanne). 2026 Jan 7;12:1733201. doi: 10.3389/fmed.2025.1733201 (PMC12819758; doi:10.3389/fmed.2025.1733201)
Supplement: Supplementary file 1 [file Table_1.docx]

**Supplementary Table S1. Complete Search Strategies for All Databases**

| **Database** | **Search Block** | **Search Terms** |
| --- | --- | --- |
| **PubMed/MEDLINE** | #1 Surgical Education | "Education, Medical, Graduate"[Mesh] OR "Clinical Competence"[Mesh] OR "surgical education"[Title/Abstract] OR "surgical training"[Title/Abstract] OR "residency training"[Title/Abstract] OR "surgical skills"[Title/Abstract] |
|  | #2 Simulation | "Simulation Training"[Mesh] OR "Computer Simulation"[Mesh] OR "Virtual Reality"[Mesh] OR "simulation-based training"[Title/Abstract] OR "simulation training"[Title/Abstract] OR "virtual reality"[Title/Abstract] OR "simulator"[Title/Abstract] OR "box trainer"[Title/Abstract] OR "laparoscopic trainer"[Title/Abstract] OR "surgical simulator"[Title/Abstract] |
|  | #3 Obstetrics and Gynecology | "Gynecology"[Mesh] OR "Obstetrics"[Mesh] OR "Laparoscopy"[Mesh] OR "Hysterectomy"[Mesh] OR "Gynecologic Surgical Procedures"[Mesh] OR "gynecology"[Title/Abstract] OR "gynecological"[Title/Abstract] OR "obstetrics"[Title/Abstract] OR "obstetric"[Title/Abstract] OR "laparoscopy"[Title/Abstract] OR "laparoscopic"[Title/Abstract] OR "hysterectomy"[Title/Abstract] OR "vaginal surgery"[Title/Abstract] OR "robotic surgery"[Title/Abstract] OR "minimally invasive surgery"[Title/Abstract] |
|  | #4 Study Design | "Randomized Controlled Trial"[Publication Type] OR "Controlled Clinical Trial"[Publication Type] OR "randomized"[Title/Abstract] OR "randomised"[Title/Abstract] OR "controlled trial"[Title/Abstract] OR "comparative study"[Title/Abstract] OR "prospective study"[Title/Abstract] |
|  | #5 Combined | #1 AND #2 AND #3 AND #4 |
| **Embase** | #1 Surgical Education | 'medical education'/exp OR 'clinical competence'/exp OR 'surgical education':ti,ab OR 'surgical training':ti,ab OR 'residency training':ti,ab OR 'surgical skills':ti,ab |
|  | #2 Simulation | 'simulation'/exp OR 'computer simulation'/exp OR 'virtual reality'/exp OR 'simulation-based training':ti,ab OR 'simulation training':ti,ab OR 'virtual reality':ti,ab OR 'simulator':ti,ab OR 'box trainer':ti,ab OR 'laparoscopic trainer':ti,ab OR 'surgical simulator':ti,ab |
|  | #3 Obstetrics and Gynecology | 'gynecology'/exp OR 'obstetrics'/exp OR 'laparoscopy'/exp OR 'hysterectomy'/exp OR 'gynecologic surgery'/exp OR 'gynecology':ti,ab OR 'gynecological':ti,ab OR 'obstetrics':ti,ab OR 'obstetric':ti,ab OR 'laparoscopy':ti,ab OR 'laparoscopic':ti,ab OR 'hysterectomy':ti,ab OR 'vaginal surgery':ti,ab OR 'robotic surgery':ti,ab OR 'minimally invasive surgery':ti,ab |
|  | #4 Study Design | 'randomized controlled trial'/exp OR 'controlled clinical trial'/exp OR 'randomized':ti,ab OR 'randomised':ti,ab OR 'controlled trial':ti,ab OR 'comparative study':ti,ab OR 'prospective study':ti,ab |
|  | #5 Combined | #1 AND #2 AND #3 AND #4 |
| **Cochrane CENTRAL** | #1 Surgical Education | [mh "Education, Medical, Graduate"] OR [mh "Clinical Competence"] OR "surgical education":ti,ab,kw OR "surgical training":ti,ab,kw OR "residency training":ti,ab,kw OR "surgical skills":ti,ab,kw |
|  | #2 Simulation | [mh "Simulation Training"] OR [mh "Computer Simulation"] OR [mh "Virtual Reality"] OR "simulation-based training":ti,ab,kw OR "simulation training":ti,ab,kw OR "virtual reality":ti,ab,kw OR "simulator":ti,ab,kw OR "box trainer":ti,ab,kw OR "laparoscopic trainer":ti,ab,kw OR "surgical simulator":ti,ab,kw |
|  | #3 Obstetrics and Gynecology | [mh Gynecology] OR [mh Obstetrics] OR [mh Laparoscopy] OR [mh Hysterectomy] OR [mh "Gynecologic Surgical Procedures"] OR "gynecology":ti,ab,kw OR "gynecological":ti,ab,kw OR "obstetrics":ti,ab,kw OR "obstetric":ti,ab,kw OR "laparoscopy":ti,ab,kw OR "laparoscopic":ti,ab,kw OR "hysterectomy":ti,ab,kw OR "vaginal surgery":ti,ab,kw OR "robotic surgery":ti,ab,kw OR "minimally invasive surgery":ti,ab,kw |
|  | #4 Combined | #1 AND #2 AND #3 |
| **Web of Science** | #1 Surgical Education | TS=("surgical education" OR "surgical training" OR "residency training" OR "surgical skills" OR "clinical competence") |
|  | #2 Simulation | TS=("simulation-based training" OR "simulation training" OR "virtual reality" OR "simulator" OR "box trainer" OR "laparoscopic trainer" OR "surgical simulator") |
|  | #3 Obstetrics and Gynecology | TS=(gynecology OR gynecological OR obstetrics OR obstetric OR laparoscopy OR laparoscopic OR hysterectomy OR "vaginal surgery" OR "robotic surgery" OR "minimally invasive surgery") |
|  | #4 Study Design | TS=(randomized OR randomised OR "controlled trial" OR "comparative study" OR "prospective study") |
|  | #5 Combined | #1 AND #2 AND #3 AND #4 |
| **Scopus** | #1 Surgical Education | TITLE-ABS-KEY("surgical education" OR "surgical training" OR "residency training" OR "surgical skills" OR "clinical competence") |
|  | #2 Simulation | TITLE-ABS-KEY("simulation-based training" OR "simulation training" OR "virtual reality" OR "simulator" OR "box trainer" OR "laparoscopic trainer" OR "surgical simulator") |
|  | #3 Obstetrics and Gynecology | TITLE-ABS-KEY(gynecology OR gynecological OR obstetrics OR obstetric OR laparoscopy OR laparoscopic OR hysterectomy OR "vaginal surgery" OR "robotic surgery" OR "minimally invasive surgery") |
|  | #4 Study Design | TITLE-ABS-KEY(randomized OR randomised OR "controlled trial" OR "comparative study" OR "prospective study") |
|  | #5 Combined | #1 AND #2 AND #3 AND #4 |
| **CINAHL** | #1 Surgical Education | (MH "Education, Medical, Graduate") OR (MH "Clinical Competence") OR TI "surgical education" OR AB "surgical education" OR TI "surgical training" OR AB "surgical training" OR TI "residency training" OR AB "residency training" OR TI "surgical skills" OR AB "surgical skills" |
|  | #2 Simulation | (MH "Simulation") OR (MH "Virtual Reality") OR TI "simulation-based training" OR AB "simulation-based training" OR TI "simulation training" OR AB "simulation training" OR TI "virtual reality" OR AB "virtual reality" OR TI "simulator" OR AB "simulator" OR TI "box trainer" OR AB "box trainer" OR TI "laparoscopic trainer" OR AB "laparoscopic trainer" OR TI "surgical simulator" OR AB "surgical simulator" |
|  | #3 Obstetrics and Gynecology | (MH "Gynecology") OR (MH "Obstetrics") OR (MH "Laparoscopy") OR (MH "Hysterectomy") OR TI gynecology OR AB gynecology OR TI gynecological OR AB gynecological OR TI obstetrics OR AB obstetrics OR TI obstetric OR AB obstetric OR TI laparoscopy OR AB laparoscopy OR TI laparoscopic OR AB laparoscopic OR TI hysterectomy OR AB hysterectomy OR TI "vaginal surgery" OR AB "vaginal surgery" OR TI "robotic surgery" OR AB "robotic surgery" OR TI "minimally invasive surgery" OR AB "minimally invasive surgery" |
|  | #4 Study Design | (MH "Randomized Controlled Trials") OR (MH "Controlled Clinical Trials") OR TI randomized OR AB randomized OR TI randomised OR AB randomised OR TI "controlled trial" OR AB "controlled trial" OR TI "comparative study" OR AB "comparative study" OR TI "prospective study" OR AB "prospective study" |
|  | #5 Combined | #1 AND #2 AND #3 AND #4 |
| **ERIC** | #1 Surgical Education | DE "Medical Education" OR TI "surgical education" OR AB "surgical education" OR TI "surgical training" OR AB "surgical training" OR TI "residency training" OR AB "residency training" OR TI "surgical skills" OR AB "surgical skills" OR TI "clinical competence" OR AB "clinical competence" |
|  | #2 Simulation | DE "Simulation" OR TI "simulation-based training" OR AB "simulation-based training" OR TI "simulation training" OR AB "simulation training" OR TI "virtual reality" OR AB "virtual reality" OR TI "simulator" OR AB "simulator" OR TI "box trainer" OR AB "box trainer" |
|  | #3 Obstetrics and Gynecology | TI gynecology OR AB gynecology OR TI obstetrics OR AB obstetrics OR TI laparoscopy OR AB laparoscopy OR TI hysterectomy OR AB hysterectomy OR TI "vaginal surgery" OR AB "vaginal surgery" OR TI "robotic surgery" OR AB "robotic surgery" |
|  | #4 Combined | #1 AND #2 AND #3 |
| **ClinicalTrials.gov** | Condition or disease | gynecology OR obstetrics OR laparoscopy OR hysterectomy OR gynecologic surgery |
|  | Other terms | simulation training OR simulation-based training OR virtual reality OR simulator OR box trainer OR surgical education OR surgical training |
|  | Study type | Interventional Studies (Clinical Trials) |

**Note:** Search strategies for Embase, Cochrane CENTRAL, Web of Science, Scopus, CINAHL, ERIC, and ClinicalTrials.gov were adapted from the PubMed strategy according to the specific syntax requirements of each database. All searches were conducted from database inception to December 2024. No language restrictions were applied during the initial search.

**Abbreviations:** MeSH, Medical Subject Headings; ti,ab, title and abstract; ti,ab,kw, title, abstract and keywords; TS, Topic Search; TITLE-ABS-KEY, title, abstract and keywords; MH, MeSH Headings; TI, Title; AB, Abstract; DE, Descriptor.
